# A water-based training program that include perturbation exercises to improve stepping responses in older adults: study protocol for a randomized controlled cross-over trial

**DOI:** 10.1186/1471-2318-8-19

**Published:** 2008-08-17

**Authors:** Itshak Melzer, Ori Elbar, Irit Tsedek, Lars IE Oddsson

**Affiliations:** 1 Physical Therapy Department, Faculty of Health Sciences, Ben-Gurion University, Israel; 2 The hydrotherapy center in Shaa'r Ha-Negev, Israel; 3 Sister Kenny Rehabilitation Institute, Sister Kenny Research Center. Minneapolis, MN, USA

## Abstract

**Background:**

Gait and balance impairments may increase the risk of falls, the leading cause of accidental death in the elderly population. Fall-related injuries constitute a serious public health problem associated with high costs for society as well as human suffering. A rapid step is the most important protective postural strategy, acting to recover equilibrium and prevent a fall from initiating. It can arise from large perturbations, but also frequently as a consequence of volitional movements. We propose to use a novel water-based training program which includes specific perturbation exercises that will target the stepping responses that could potentially have a profound effect in reducing risk of falling. We describe the water-based balance training program and a study protocol to evaluate its efficacy (Trial registration number #NCT00708136).

**Methods/Design:**

The proposed water-based training program involves use of unpredictable, multi-directional perturbations in a group setting to evoke compensatory and volitional stepping responses. Perturbations are made by pushing slightly the subjects and by water turbulence, in 24 training sessions conducted over 12 weeks. Concurrent cognitive tasks during movement tasks are included. Principles of physical training and exercise including awareness, continuity, motivation, overload, periodicity, progression and specificity were used in the development of this novel program. Specific goals are to increase the speed of stepping responses and improve the postural control mechanism and physical functioning. A prospective, randomized, cross-over trial with concealed allocation, assessor blinding and intention-to-treat analysis will be performed to evaluate the efficacy of the water-based training program. A total of 36 community-dwelling adults (age 65–88) with no recent history of instability or falling will be assigned to either the perturbation-based training or a control group (no training). Voluntary step reaction times and postural stability using stabiliogram diffusion analysis will be tested before and after the 12 weeks of training.

**Discussion:**

This study will determine whether a water-based balance training program that includes perturbation exercises, in a group setting, can improve speed of voluntary stepping responses and improve balance control. Results will help guide the development of more cost-effective interventions that can prevent the occurrence of falls in the elderly.

## Background

Postural control is the foundation of our ability to stand, move independently or prevent fall during loss of balance when fall is initiated. Thus, deterioration of the postural control system due to aging can lead to balance impairment, inability to react properly (e.g., speed and amplitude), limitations of mobility and eventual disability. Consequently, there is a clinical need for developing cost-effective interventions for improving balance and reducing balance impairments that may lead to falls. In the elderly population, about one out of three individuals fall [1]. Falls may result in acute injuries, including traumatic brain injuries [2] and spinal cord injuries [3] or hip fractures [4]. Hip fractures occurring as a result of falls are a common source of impairment, disability and even death [5]. Overall, fall related injuries constitute a serious public health problem and are associated with high health care costs [6].

There are well-documented studies that show that exercise is beneficial to people with heart disease [7] and that it reduces the risk of diabetes [8], stroke [9], hypertension [10], and osteoporosis [11], increases muscle power [12], reduces frailty [13], and improves balance control [14-16] in the elderly. Research studies investigating exercise as a means of falls prevention in older adults has shown controversial results. Several studies show that exercise prevents falls [17-22] and other studies have shown no effect [23-25]. Even well-respected studies on balance training in the elderly population appear to have either ignored or misunderstood the importance of basic principles of physical training and exercise physiology (e.g., awareness, continuity, motivation, overload, periodicity, progression and specificity) [26-30], especially with respect to the concept of specificity in balance training in the elderly population. Thus, an exercise intervention targeting a certain function must provide a challenge/overload to the system and be progressive as well as specific for this function, otherwise a training effect should not be expected. A common misunderstanding appears to be that strength or even cardiovascular training per se should improve balance function [31-37]. There is a documented relationship between falls and muscle strength in the elderly [32]; however, results are controversial and other studies show minimal or even no differences in strength between fallers and non-fallers [38,39]. Studies that have demonstrated improvements in balance function after strength training have usually related balance to a task where muscle strength, or joint torque production, is a dominant and obvious component of the task (e.g., sit-to stand [40,41], rapid backward walking [34]). In fact, balance improvement related to strength training appears to occur primarily in individuals with severely compromised strength and muscle function [37,42,43]. This view is supported in a Cochrane Review by Latham et al. [44] of 62 randomized control trials including a total of 3674 subjects showing no statistically significant effects of progressive strength training in elderly subjects on objective clinical measures of balance function or on physical disability measures. According to the principles of training, some of which were introduced above, training of balance as a skill must incorporate exercises that closely mimic and provide a challenge to the successful performance of functional tasks. This is supported in the literature; in fact, it has been reported that exercises functionally related to balance, mobility, postural alignment and coordination [45] that challenge the postural control system [45,46] can improve the ability of fallers to maintain their balance. Furthermore, it has been suggested that a routine of Tai Chi exercise can help improve [47] or maintain [48] balance control. Using a balance-specific intervention that included perturbation exercises, Wolfson et al. [48], in an elegant study, were able to demonstrate improvements in balance function in healthy 75–90-year-old individuals that appeared to counteract normal age-related balance deterioration. Recently, Mansfield et al. [49] suggested the use of a perturbation platform that moves suddenly and unpredictably in one of four directions: forward, backward, left or right as a part of a training program. Oddsson et al. [50] proposed a specific training program that involves use of unpredictable, multi-directional perturbations to evoke stepping responses in elderly persons. A randomized controlled trial of the two above-mentioned studies has been conducted to investigate effects of the proposed training programs on gait and balance function in healthy elderly individuals (not yet published). In an interesting training approach for adults, Rogers et al. [51] showed that a three-week period of either voluntary or waist-pull-induced step training reduced step initiation time. During the three-week training regimen, the subjects performed either twice weekly induced step training (destabilizing large waist pulls) or voluntary step practice to a somatosensory reaction stimulus cue (nondestabilizing small waist pulls). However, three of the above-mentioned studies [48,49,51] utilized expensive equipment (e.g., moving-platform and waist pulls) that would not be available to most elderly individuals or even in rehabilitation clinics. The currently proposed water-based intervention is based on Oddsson et al.'s [49] method and will incorporate similar principles in a group setting with the use of inexpensive equipment that can be acquired by anyone in the pool in a group setting.

A rapid step is the most important protective postural strategy since it can prevent a fall from occurring. It can arise from large perturbations (e.g., slips, trips and collisions), but is also frequently recruited at lower magnitudes of perturbation or as a consequence of volitional movement (e.g., self-induced perturbation such as turning, bending, reaching) [52]. It is important for fall-prevention programs to include specific stepping exercises on balance-recovery reactions, whether they are compensatory or voluntary in nature. A recent study Melzer et al. [53] found that voluntary step execution was statistically significantly different between non-fallers and fallers during dual task condition (adding a cognitive load). The risk of falling by participants with dual task voluntary step execution times ≥ 1100 ms was 5 times that of participants with execution times <1100 ms. Thus, improving the ability to step quickly, especially under dual task conditions, in response to a loss of balance determines whether a fall occurs in elderly persons. Compared to young adults, older people showed reduced step length [54] an increased frequency of collisions between the swing foot and stance leg during lateral perturbations [55] and an increased frequency of multiple-step responses [54-56], with a lateral second step following the forward or backward step [56]. All the above results suggest that specific and progressive training to improve the speed and length of the stepping response may reduce the risk of falls in older subjects.

Volitional or compensatory stepping can be divided into three phases: 1) the step initiation phase, 2) the anticipatory postural adjustments phase (APA), and 3) the swing phase. The duration of the step initiation phase is mainly dependent on peripheral sensory detection and afferent nerve conduction time followed by central neural processing and efferent nerve conduction time.

Step training (volitional or compensatory) may increase the speed of central neural processing capabilities during the specific function. During the APA phase, neural control of volitional stepping differs from stepping evoked by postural perturbation [52,57], whereas these APAs are typically absent or significantly reduced during large perturbations [52,58,59]. Thus it was suggested that training volitional stepping might lead to improved control of the APAs, but would probably not improve compensatory stepping, especially due to lack of training specificity. Finally, the swing phase incorporates the actual motor execution of the task when the leg is lifted and moved to the target location. Hence, specific and progressive training using water resistance to improve the swing phase speed may provide a benefit in the ability to execute not only effective compensatory stepping reactions but also volitional stepping response.

The present study aims to test a novel water-based training program that includes perturbation of balance during water-based exercises, specifically targeting stepping responses. To date, only a few studies have examined the effects of water-based training on balance control in the elderly. These studies demonstrated increased Berg Balance score [60], improved leaning balance [61,62] and Functional Reach [63], and improvement in the step test (step 7.5 cm high and return to the floor as many times as possible over a period of 15 seconds), quality of life, but not fear of falling [64], following training. To our knowledge, no studies have directly addressed the potential of using perturbation-based training to counter specific impairments in compensatory and voluntary stepping responses. By training in a water environment, the fear of falling as a result of perturbation exercises may be reduced in elderly persons. Fear of falling may reduce the feasibility of land-based perturbation training (i.e., outside the water). We hypothesize that subjects who undergo a water-based training program that includes perturbation exercises will show greater improvements in the ability to step rapidly, compared to control subjects who undergo no training. In this paper, we describe the development of a water-based training program that includes perturbation exercises and lay out the protocol for a randomized controlled cross-over trial to evaluate the efficacy of this program.

### Development of the water-based training program

#### Water-based perturbation exercises

While subjects try to stand in the water and maintain a stable upright stance over the base of support (BOS) – defined by the feet – water movement and turbulence play an important role by overloading the postural control systems during standing and reaching movement (while feet are fixed on the pool floor) and during change of support movement (e.g., stepping). For stance (exercise levels 1 and 2 in the proposed water-based training program), this relative motion of water causing displacement of either the body's Center of Mass (COM) (via water motion and turbulence) or the BOS (standing on unstable balls or a "noodle" placed underneath the subject's feet on the pool floor), these exercises in water might cause perturbed balance (e.g., during leaning, turning, reaching) thus challenging the balance control system. During gait exercises (exercise levels 3 and 4 in the proposed water-based training program), the water motion and turbulence can also be induced and create disturbance due to unexpected turbulence and water resistance perturbation of the COM (e.g., simulate tripping) and BOS perturbations (e.g., simulate slipping due to a slip on the pool floor on a low-friction pool surface, tripping on an obstacle placed on the pool floor). Additional exercises (level 5) will use perturbation exercises provided by the instructors or classmates to evoke balance-recovery stepping reactions against the water resistance. The perturbation methods may fulfill the fundamental biomechanical requirement (disruption of the COM-BOS relationship) and elicit postural and stepping reactions that are similar in many respects to land-based training. Hence, it is possible that the training benefits derived using water-based training that includes perturbation exercises may generalize to the reactions evoked outside the water. This, however, remains to be established since postural reaction within the water may be different from postural reaction on land.

For this study, the motion of the water inside the swimming pool and the water turbulence were made specifically to deliver the postural perturbations needed to improve postural control mechanism in up-right stance. To evoke stepping and grasping reactions during training, additional pushes will be made by the instructors and by the training associates. Subjects will be instructed to respond to the pushes by stepping as quickly as possible, if required. One important feature of the perturbation approach is that the direction of perturbation (pushes) will be unpredictable.

### The water-based perturbation training program

The water-based training program is based on the Functional and Specific Balance Training Program (Five Level Program) published by Oddsson et al. [49]. The training program adheres to the principles of exercise prescription – specificity, progressive overload, individualization. The program is performed on five levels, where each level reflects different demands on the postural control system. Levels 1–4 include exercises that are focused on the skill to maintain balance (voluntary control), whereas level 5 also includes perturbation exercises that focus on the skill to recover balance (automatic postural responses). We have adapted the program to water-based training.

**Figure 1:**
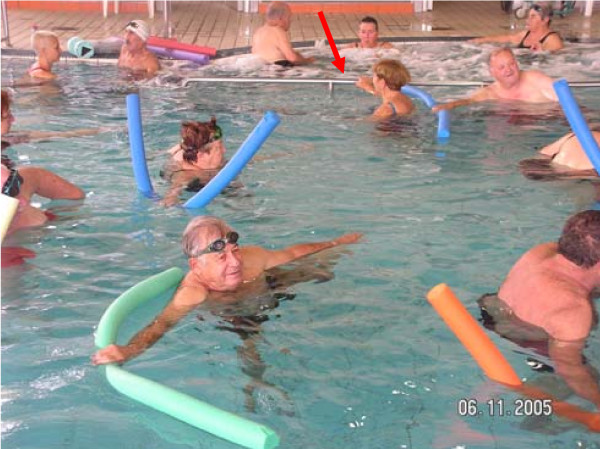
Level 1 exercise – standing on a noodle, maintaining balance with external support (see the red arrow).

**Level 1**: Standing exercises with external support (e.g., pool wall) (Figure 1).

Exercises on this level may present little challenge to the postural control system. The goal of the training at this level is mainly directed towards a cognitive understanding of the exercises, and an improvement in self-confidence for the water-based exercises on higher levels. At this level the participants also adjust to the water, become aware of unique characteristics such as the floating force and turbulence, the warmth and feel of the water. Participants begin with simple exercises such as diving, breathing and holding their breath under the water, and gaining confidence in this relatively new medium. These exercises can also be used as safe "rest" after more difficult bouts of exercise. Some general effects on strength, coordination, and conditioning are expected. Everyone should progress through this level as soon as possible. However, elements from exercises at this level will also be included on the other levels.

Examples:

1. Standing and holding the pool wall, wide stance, with support of one hand on the wall or a fixed object. Repeat with narrow stance and support.

2. Standing and holding the pool wall, wide stance, with support, and shift weight left and right, forward and backward as far as possible.

3. Standing and holding the pool wall and rotating trunk left and right as far as possible, with support.

4. Standing and holding the pool wall, wide stance, with support, lift one foot at a time. Repeat in narrow stance.

5. Standing and holding the pool wall, wide stance, with support, and inserting their head into the water.

6. Changing the base of support or closing eyes will increase the level of the exercises.

**Figure 2:**
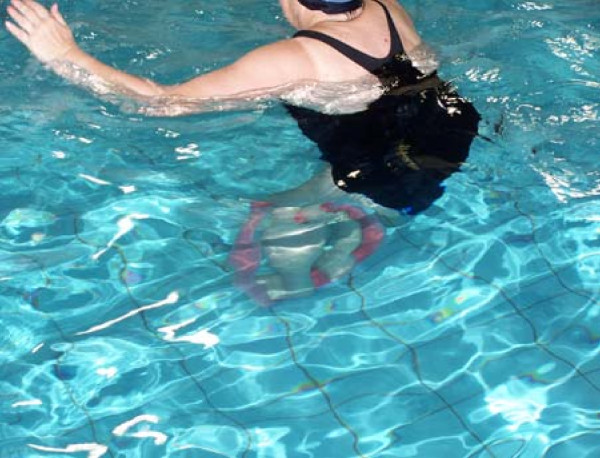
Level 2 exercise – standing on a flat ball with narrow base stance, maintaining balance without external support (e.g., paying attention to how challenging the task is).

**Level 2**: Standing exercises including double leg stance with no external support (Figure 2).

The training on this level will challenge postural control in a predictable and controlled manner through voluntary movements. Exercises are similar to level 1 but without external support. For the purpose of activating relevant associated postural adjustments, which are the target of the training at this level, it is more beneficial to execute an exercise slowly with small amplitude rather than using external support, because this will completely change the set of muscles that are recruited for balance control during the task. Thus, it is expected that water motion (small unexpected perturbations) will challenge postural control, increase lower limb muscle activity and might be very challenging for elderly persons. Depending on the skill of the subject, these exercises can also be used as safe "rest" after more challenging bouts of exercise.

Examples: the same as in level 1 but without external support (holding the pool wall).

**Level 3**: Standing exercises including single leg stance with no external support.

To increase the challenge of exercises at this level, instructors will add dual task exercises and external resistance exercises (e.g., weights).

Examples:

1. Standing on one leg while the other leg pushes and pulls a "noodle" (long float).

2. The same as the first, while playing with a ball or adding cognitive task.

3. Standing on wide/narrow base of support, holding a "noodle" with both hands and trying to push it in the water while maintaining balance.

4. Riding on a "noodle" and maintaining balance.

5. Standing with one leg on a balance ball or "noodle".

6. Standing with both legs on the "noodle" and maintaining balance, with or without an additional task

7. Standing with both legs on a "noodle", holding a "noodle" with both hands and trying to push it in the water while maintaining balance.

**Level 4**: Gait exercises, dynamic base of support with no external support. Exercises on this level incorporate specific gait training against water resistance.

Examples:

1. Walking in all directions (e.g., forward, backward and sideways), at different speeds and in different water depths.

2. Changing direction as fast as the subject can.

3. Walking on a "noodle" or flat balls with and without additional cognitive tasks.

4. Adding head movements to challenge the vestibular system.

**Figure 3:**
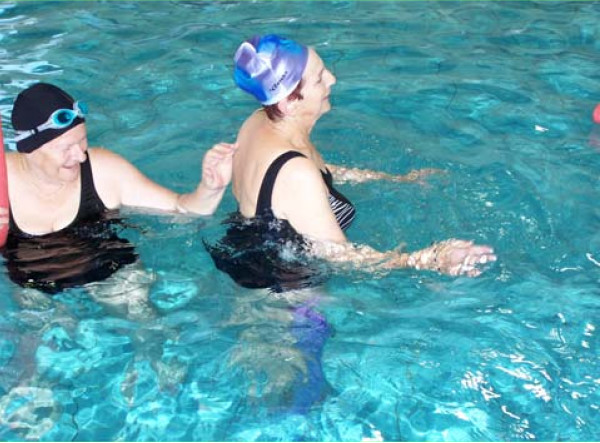
Level 5 exercise – standing on a noodle and maintaining balance while the participant is being pushed.

**Level 5**: Perturbation exercises, for improving reactive and proactive responses. Exercises on this level include different forms of external perturbations, expected as well as unexpected ones that require a proactive or reactive response by the subject. Perturbations requiring a proactive response by the subject may be applied during different forms: water turbulence, the partner or group exercises (Figure 3).

Examples:

1. The same as levels 3 and 4 but against massive water turbulence and in deeper water.

2. Standing with wide stance while his or her partner pushes the classmate in different directions, with and without warning.

3. The same as before but while standing on noodle, changing base of support, in eyes open/closed condition, adding cognitive tasks.

4. Walking and being pushed by instructor or classmates with and without additional cognitive tasks.

Participants will also be exposed to exercises where they will be encouraged to either resist a balance perturbation or avoid stepping or executing one or several steps as quickly as possible, including forward and immediately lateral.

#### Specificity

The need to target specific goals is considered an important concept, well established and accepted in the exercise physiology literature for decades [26-30]; any successful athlete must live by these rules to maintain or improve function and performance. These fundamental principles of successful exercise prescription apply to anyone regardless of age, sex, or level of fitness [65], even patients.

The water-based program that includes perturbation exercises targets specific age-related impairments in compensatory and voluntary stepping responses, and water motion and turbulence during standing and walking exercises targeting balance control system to allow keeping balance as follows:

1. **Speed and length of stepping: **Due to the reduction in speed [53,66] and length [54] of voluntary stepping, older adults take more steps to recover balance [55] and tend to respond to perturbations with a multiple-step response more frequently than younger adults [54-56]. Although use of multiple steps can be a pre-planned strategy in some situations [54], it appears that the multiple steps emerge as a consequence of lack of lower limb muscle power and its relation to reduced functional abilities, which is well documented [67], and inability to take a rapid and long first step. To increase the ability to take a rapid and long step, subjects will be instructed to respond to the perturbation (level 5 exercises) as quickly with as long a step as they possibly can.

2. **Medio-Lateral instability during standing and stepping**: Medio-lateral instability in elderly individuals compared with young and elderly fallers compared with nonfallers [39,68] is well documented. To increase the ability to control Medio-lateral balance during stance, subjects will be instructed not to take a step and remain standing during standing exercises (level 2 and 3 exercises) against water turbulence, and will be challenged by narrow base and tandem stance or even more difficult standing on flat ball or a "Noodle". This specific training will improve the balance control mechanism.

3. **Medio-Lateral instability during stepping: **Medio-lateral instability in elderly individuals compared with young [52,59] and the tendency of elders to fall laterally toward the swinging leg during compensatory step execution appears to be a problem related to increased risk of lateral fall and hip fracture [55]. To increase ability to take an additional long lateral step rapidly, subjects will be instructed to respond to the perturbation (level 5 exercises) as quickly as possible by stepping forward (or backward) and adding an extra lateral step as quickly as they can. This exercise resembles plyometric exercises which improve muscle power and speed.

4. **Improved cross-over stepping: **Maki et al. [55] suggested that collisions between the swing foot and stance leg during lateral perturbation can delay speed of stepping and increase risk of fall. Water-based stepping exercises against water resistance is a tool to train and increase the speed of stepping rapidly with no fear that such collisions will cause a fall during exercise. Also during walking exercises (level 4 exercises) lateral cross-over steps will be made (also over an obstacle that will be placed on pool floor, under the water and against water resistance).

5. **Reduced ability to take a rapid step during attention demanding tasks (dual task): **Balance-recovery reactions require attentional resources and cognitive processing during compensatory [69-71] and voluntary stepping [53,66,72]. To improve the ability to rapidly switch attention, cognitive tasks will be included during training.

6. **Increased visual dependency during control of posture: **During some of the exercises in each of the training levels (levels 1–5), subjects will be instructed to close their eyes and control balance in stance and during water turbulence (i.e., water perturbations) subjects will be instructed to keep their body rigid.

#### Overload and Progression

The main factor that modifies the demand of the task is water resistance. Aquatic physical therapy is based on several important bioengineering properties. The basic forces acting upon the patient while in the water consist of buoyancy, drag, and inertial forces. According to key hydrodynamic principles, a moving limb under water is acted on by drag and lift forces. Total drag can be defined as a resistant force opposite to the direction of movement of an object. The viscous properties of the water behind an object are referred to as form drag. The turbulence is generated by the mass of water that is sucked along behind an object in the form of retarding eddies and is proportional to the size and shape of the frontal area that is being pushed against the water. The relationship between the drag force and the velocity of the movement is nonlinear, so that the drag increases as a function of velocity squared (v^2^). Once the speed doubles, the consequent drag force quadruples. If the participant is in a head-out immersion, the buoyancy decreases his weight-bearing on the ground and by that his sensory input is reduced; this may increase the challenge on the balance control system, thus increasing overload. Furthermore, when the water is turbulent, it causes a continuous perturbation of posture during fix of support exercises and especially during exercise involving change of support (e.g., stepping, walking). Progressions will also be made by including open and closed eyes, stance width, single (level 3) and double leg support (level 2), standing on unstable support surface (such as a flat ball and noodles) during standing. Increased step speed will dramatically increase water resistance and a rapid extra step after the first step is challenging for power production (at all levels of training). Increasing the magnitude of the applied perturbations (e.g., pushes by the instructor and classmates) and applying it unexpectedly, and increasing the speed of forward and backward walking increases the load during gait and balancing.

#### Individualization

The balance training intervention is performed on five levels where each level reflects different increasing demands on the postural control system Training programs should be tailored to the individual needs and abilities of the participant [73]. This process requires interaction with an experienced therapist, instructor, or coach and should not be based on a "cookbook" approach to training. As in any type of training, for improvement to occur it is crucial to maintain a progressive and specific training load for each of the participants. Progressions should be made when the individuals have reached adaptation. On each level the instructor can instantly modify an exercise to be more or less challenging for each participant. For example, the instructor can increase the difficulty of a certain exercise by instructing a participant to use less external support, close the eyes, decrease the support area (stand on one leg, narrow the stance or on noodle, not use the arms to balance, move the head during the exercises, etc.) while another participant will not find this equally challenging. These "tools" allow the instructor to implement exercises on a group level that are still challenging for each individual, even if the skill level in the group varies. In addition, variability is important to keep subjects motivated and excited to pursue the training.

#### Generalizability

The goal of each session in the current program is to constantly challenge the postural control system with exercises that incorporate elements related to the demands of normal activities of daily living [74]. To promote generalizability, during the water-based and perturbation exercises the subjects perform a variety of real life dual tasks (cognitive and movement tasks).

#### Instructors

The program should be implemented by two instructors or physical therapists (and a life saver outside the pool) who, based on each individual subject's background and current skill/fitness level, can assess at what level a subject should begin the training program to maximize compliance and outcome and maintain safety. Any form of physical training, either learning and perfecting a sport skill or rehabilitating after an injury or disease, is a process that incorporates both physiological and psychological factors. These factors must harmonize on an individual level for the training process to be successful. A very important role of the instructor is to monitor the training process on an individual level and adapt the training program to optimize outcome and maximize performance. When needed, the instructor should be able to modify the execution of an exercise "on-the-fly" to customize the difficulty on an individual level. The instructor and the lifeguard are near by and fully alert to the security of each participant.

The instructors should be able to direct the subject's attention toward important aspects of the skill being learned to increase awareness. To enhance learning, subjects in this study are provided with instructions regarding the task goals. For example, subjects will be told to take steps as quickly and for as long as possible during balance perturbations or to avoid limb movements during upright stance counteracting water turbulence. These instructions will be gradually reduced as training progresses to avoid dependency [75]. During the training period there is little risk that a participant will fall. Although the participants will be pushed during the perturbation exercises, the water will keep the participant from falling on the pool floor and injuring him/herself in the most demanding levels of exercise. The risk should be minimal. At the beginning, exercises in level 5 will incorporate mild/gentle external balance perturbation exercises applied by the instructor. **It is important to note that these exercises will be customized to each subject's ability. They will be designed to be challenging but never dangerous**. The participants will be repeatedly instructed to ask for help if they feel uncomfortable about performing a certain exercise. Initially, the training will progress slowly to help everyone find their level of ability and not take any unnecessary risks. In fact, an important part of the training is to make each individual learn what their ability is. Overall, the training should be less dangerous than land-based exercises or other activities of daily living.

#### Improving gait and balance in Stroke survivors – Pilot Data

The following section describes findings from a pilot study conducted in collaboration between Ben-Gurion University and the hydrotherapy center in Shaa'r Ha-Negev using the training program presented here. A group of 8 participants (3 female, 5 male) between 71 and 85 years participated in the program two-times a week for 12 weeks. Compliance with the exercise program was excellent (88% average attendance over the 12-week program). Overall, functional status variables improved for all of the participants. Stroke survivors showed the largest improvement in Timed Up and Go (from 14.3 sec to 12.2 sec), and in Berg Balance Test (from 47.3 to 50.2).

## Methods/design

### Experimental protocol to assess the efficacy of the training program

A randomized controlled cross-over trial will be performed to assess the efficacy of the water-based training program that uses perturbation of balance to trigger automatic reflex-like postural responses as well as volitional stepping reactions. Ethical approval for this study was obtained from the Research Ethics Board of Soroka Medical Center and Ben-Gurion University Helsinki Committee (Trial registration number #NCT00708136).

### Recruitment of subjects

Community-dwelling older adults will be recruited from the community of the Sha'ar Hanegev council via advertisements in local newspapers, the internal brochure of the "Yahdav" elderly center and by personal contacts. The subjects who ambulate independently will be randomly allocated to either the water-based exercise group or the control group without intervention. A short interview examined whether the subjects met the inclusion-exclusion criteria. Participants were excluded if they had received physiotherapy or hydrotherapy, or attended community exercise classes in the past six months, had orthopedic surgery within the prior year; showed an indication of cognitive impairment (Mini-Mental Score <24 [76]), had severe focal muscle weakness or paralysis, serious visual impairment, severe peripheral or compression/entrapment neuropathies, any neurological disorders causing balance or motor problems, or cancer (metastases or under active treatment). Prior to their inclusion all subjects received medical clearance from their primary care physician to participate in the study.

### Randomization and blinding

Subjects will be randomly assigned, using a table of random numbers, to either the balance training intervention group or the control group, who received no intervention. Random sequence generation will be performed by an individual who will not interact with subjects during the balance-testing sessions (IM). Subjects will be informed that they will be randomly assigned to one of the two groups, and the control subjects will be offered the opportunity to participate in a separate 3-month water-based training program after the training period. The individual who administers the training programs (OE) will be the only member of the research team aware of the subjects' group allocation. A blinded research assistant will administer the balance tests and will perform any data processing that involves subjective judgments. Scripts will be used during testing to ensure that all subjects receive the same instructions.

### Intervention

The intervention program will last 12 weeks, with two 30-minute sessions per week (24 training sessions). This duration and intensity is similar to that used previous perturbation-based training studies [50,51,77,78]. All subjects will be asked to refrain from initiating any other new exercise programs, or otherwise consciously change their activity levels, during their participation in the study. For the perturbation-based training program, each session will consist of 2–5 minutes of warm up exercises, 15–20 minutes of water-based exercises including voluntary step training (see levels 1–4 exercises), about 5 minutes of perturbation training, and 2–5 minutes of cool-down exercises.

### Types of data to be obtained and measurement techniques

The following tests will be performed, for research purposes only, on all participants in the study. All outcome measures will be assessed before and after the training period.

#### Functional and objective balance tests

The following standardized functional tests will be administered.

**Figure 4:**
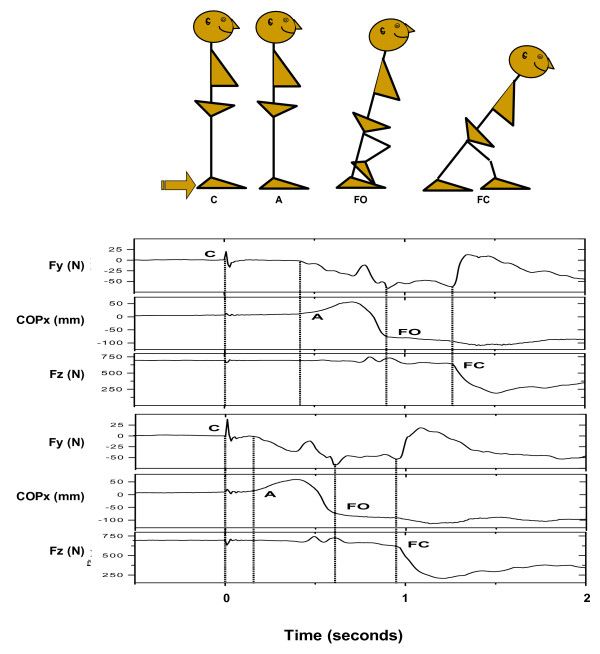
An example of forward step execution data for a 90-year-old subject during single (bottom) and dual (top) task conditions. Note that the tap cue is detectable in all of the signals. Note the differences in step reaction times between the two task conditions (distance between C and A). COPx = Mediolateral center of pressure, Fy = Ground reaction forces (shear forces) in antero-posterior direction. Fz = Vertical ground reaction forces, mm = millimeter, N = Newton. The following events are marked with vertical lines: Tap cue (C), Initial deviation of COPx (A), Foot-off (FO), Foot contact (FC).

1. Voluntary Step Execution Test[53,66,72]: This test will be performed on the portable Kistler 9287 force plate (Kistler Instrument Corp, Winterthur, Switzerland). Participants will be instructed to stand relaxed, view a target placed on a wall 3 m in front of them and to take a step as quickly as possible following a random somatosensory cue. Following a brief learning period, assessments will be made in three directions in random order (forward, backward, and sideways). The time from lifting of the foot (step start) to placing it on the ground will be measured using a portable force plate. The average of these measurements will represent the step execution time. Step execution will also be measured during performance of a secondary attention-demanding task, as described in details by Melzer and Oddsson [53,66,72] (Figure 4). Inter- and intra-rater reliability was good to excellent for the pooled population of young and elderly and for elderly only ((ICC(2,1) = 0.74–0.92 and 0.62–0.88, respectively) [72].

The five primary outcome measures correspond to the specific aspects of voluntary step execution that were targeted in the water-based training program that include perturbation exercises. The following events were extracted from the ground reaction force data (cf. Figure): (1) The step initiation phase was detected from the tap cue (a spike in the shear ground reaction forces in the anterior-posterior direction (C in Figure) to the first medio-lateral deviation of the center of pressure (COP) towards the swing leg (COP excursion greater than 4 mm from baseline sway following the tap (A in Figure); (2) Foot-off (FO in Figure 4 was defined at the sudden change in the slope of COP towards the stance leg in the medio-lateral direction; (3) Foot-contact (FC in Figure 4) was defined as the onset of unloading the stance leg seen in the vertical ground reaction force; (4) Preparatory phase duration was calculated as the time from step initiation to foot-off; (5) The swing phase duration was calculated as the time from foot-off to foot-contact. The analysis of step execution data extracted specific temporal events using a program written in MatLab (Math Works Inc, Cambridge, MA, USA).

We have chosen the voluntary step execution test during single and dual task conditions to be measured since in a review published recently, Piirtola and Era [79] found that measures related to dynamic posturography (moving platforms) were not predictive of falls [79]. However Melzer et al. [53] found that application of the voluntary step execution test similar to the test in the proposed study identified elderly fallers.

2. Stabilogram-Diffusion Analysis: An automated algorithm will be used to extract standardized stabilogram-diffusion parameters from each of the COP data sets collected during quiet standing. These parameters include diffusion coefficients, critical displacement, critical time and scaling exponents for both lateral and anterior-posterior sway directions [80,81]. For each of the conditions (eyes open and eyes closed), participants will be required to stand on the platform 10 times with the eyes open and 10 times with the eyes closed for 30 s each. For each trial, they will be instructed to sway as little as possible.

**Figure 5:**
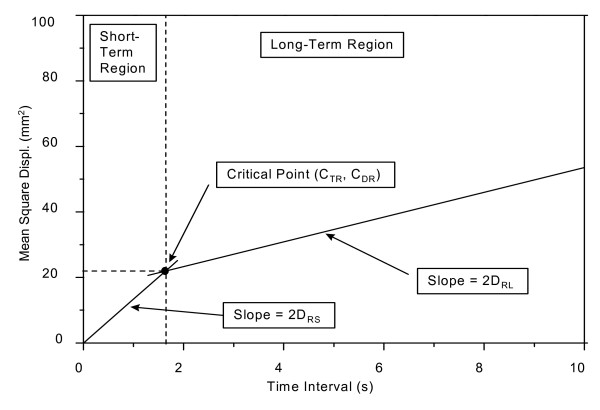
A schematic Stabilogram-Diffusion plot indicating the parameters that can be extracted to describe properties of the postural control process.

A computerized automated procedure will be used to analyze the center of pressure recordings. Eighteen parameters (see below) will be extracted from the stabilogram-diffusion analysis to describe different aspects of the postural control process. These include two sets of the following parameters; one for medio-lateral and one for the anterior posterior direction and one resultant (refer to Figure 5 for a graphical illustration of the stabilogram-diffusion plot):

C_T _Critical Time, separates short-term and long-term regions. Indicates where the balance control system switches from an open-loop to a closed loop behavior.

C_D _Critical Displacement, indicates mean square displacement of the COP at the Critical Time. Indicator of short-term body sway that the subject is able to control.

D_S _Short-term diffusion coefficient, indicates the stochastic activity (frequency and amplitude) of the COP, the random walker, in the short-term region. Assessed from the slope of the stabilogram-diffusion plot in the short-term region.

D_L _Long-term diffusion coefficient, as D_S _for the long-term region.

H_S _Short-term scaling exponent, indicates the degree of correlation between past and future values of the COP. A value of 0.5 indicates a classical random walk (50% chance of COP displacement in one direction or the other); <0.5 indicates anti-persistence, i.e., the COP tends to come back to a certain equilibrium point (suggests closed-loop control); >0.5 indicates persistence, i.e., the COP tends to drift away (suggests open-loop control). Values can be between 0 and 1.

H_L _Long-term scaling exponent, as H_S _for the long-term region.

Stabilogram-diffusion parameters will be extracted for both eyes-open and eyes-closed conditions. The difference in critical displacement between eyes-open and eyes-closed will be used as an indicator of how much an individual relies on vision to control body sway. A large increase in sway with eyes closed would indicate visual reliance.

3. Berg Balance Scale[82]: The participant is scored on 14 tasks graded on a 0–4 scale to evaluate balance function under different conditions (maximum score 56). Wang et al. [83] found good internal consistency reliability (Cronbach's alpha = 0.77), good inter-rater reliability (ICC(2,1) = 0.87), and moderate correlation with the TUG and usual gait speed (Spearman's rho = -0.53 and 0.46, respectively).

4. Get-Up-and-Go Test[84]: The participant is seated in a chair that is placed 3 meters from a wall. The participant is instructed to rise from the chair, walk at a normal pace to the wall, turn around, return to the chair, and sit down. This task is timed by the researcher using a stopwatch. A practice trial is allowed prior to the actual test. The tester remains near the participant throughout the test to prevent a fall or injury. The TUG [84] was found to be correlated with other measurements, such as gait speed (*r *= -0.61) and the Barthel Index (*r *= -0.78). The TUG was shown to have a sensitivity and specificity of 87% for identifying older adults who are prone to falls. The measures showed significant inter-correlation (r = 0.93). Intra-tester and inter-tester reliability have been reported as high in elderly populations, ICC = 0.92–0.96 [85].

#### Questionnaires

The following questionnaires will be administered to all participants enrolled in the study. Other secondary outcome measures will be analyzed to evaluate whether the training had any benefits extending beyond effects on balance reactions

1. Fear of falling (Fall Efficacy Scale, FES).

2. Memory and cognition (Folstein Mini-Mental State Examination).

3. Late Life Function and Disability Index (LL-FDI). The LL-FDI is a scale developed by Jette and Haley at Boston University. This scale is specifically designed to be sensitive to changes in physical function, something previous measures did not do as well [86,87]. The LL-FDI was recently translated to Hebrew. The function component of LL-FDI found to be a reliable (ICC(2,1) = 0.77–0.90) and valid measure of balance function (Berg Balance Scale, *r *= 0.48, *P *< 0.001) and gait speed (Get-Up-and-Go Test, *r *= -0.52, *P *< 0.001) [88].

### Randomized Cross-Over Clinical Trial

We have decided to conduct a Randomized Cross-Over Clinical Trial, a prospective, analytical, experimental study. Individuals will be randomly allocated to one of two groups (water-based balance training program and controls) and, after a 3-month training period with a 1-month washout period, participants will be switched to the other group for the same period. The control group will not receive any instructions and will not be encouraged to change their physical activity, activities of daily living or social habits during the study. The participants will be examined during the baseline and post-test in a single blind fashion to determine whether a water-based training program that includes external perturbation exercises is an effective treatment to improve speed of stepping and balance control measures in upright stance.

### Statistical analysis

Normality will be ascertained by the Shapiro-Wilk statistic (non-significant) and normal Q-Q plots, indicating that parametric statistics are able to be used. A repeated measures analysis of variance for intervention * time will performed to determine if there are any differences in effect between the two exercise training circuits. The repeated measures of time are baseline, 3 months (post-intervention of the first group – 1^st ^circuit) and (6 months post-intervention of the second group – 2^nd ^circuit). The effects of training on the mean step reaction times (step initiation, time to foot-off, time to foot-contact, preparatory phase, and swing phase), postural stability measure (C_T_, C_D_, D_S_, D_L_, H_S_, H_L_), BBS, TUG, and LL-FDI scores will be calculated using SPSS (version 10.1, SPSS Corp., Chicago, IL). Order of intervention will be also analyzed as a covariate in order to detect any carryover effect from the previous intervention. If a difference between the two training circuits will be found for any variable, *post hoc *comparisons with Bonferroni adjustment will be made. Wilcoxon signed rank test and Mann-Whitney U-tests will be used in case the variable was not normally distributed. Results will be presented as mean values ± SD, the level considered to be statistically significant was *p *< 0.05.

### Sample size estimate

A sample size requirement of 36 was estimated to detect an effect size of 0.8, powered at 80% and alpha of 5% was chosen for a clinically meaningful estimate. The estimation was performed two-sided. Based on a paper by Melzer et al. [53] the investigators found that step execution (average foot-contact times) during the execution of cognitive tasks were 1414 ms ± 417 for elderly fallers and 1165 ± 352 in elderly non-fallers. It was estimated that 32 subjects are required to detect a clinical change in this parameter. For the current study we have used an attrition rate of 10% (32 × 1.10 = 36).

## Discussion

This study is the first to use water-based training that includes perturbation exercises and water turbulence in a group setting as an intervention to reverse age-related impairments in the ability to step quickly and recover from loss of balance. It differs from previous studies done in the water in this area in a number of significant ways. To date, no study has proposed using a water-based training program that includes perturbations to train stepping reactions and investigate the potential of water turbulence to improve stability. Only four exercise regimens have proposed the use of perturbations to improve stepping in elderly persons; all were land-based exercises regimens [49-51,89].

The water-based training program that includes perturbation exercises as outlined is targeted specifically to: 1) step execution-recovery reactions that are known to be impaired in older adults and associated with increased falling risk [53]; 2) the water-turbulence during voluntary exercises challenges balance control in multiple directions; 3) the perturbations are applied by instructors and classmates in predictable and unpredictable manners that allow subjects to exercise safely and progress when needed. Based on our previous findings [53,66], ongoing cognitive exercises are included to increase the load and specificity of training.

We hypothesize that the novel water-based balance training that includes perturbation exercises will improve balance control and increase speed of stepping, and this should help to reduce the incidence of falls. Due to the limitation of randomized controlled cross-over trials, the investigation of effects on falling is beyond the abilities of the present study. The results of the present study will add evidence regarding the effectiveness of perturbation-based balance training programs, and will be novel in conducting the perturbation exercises in water against water resistance and in a group setting that provides cost-effective exercise programs for long-term improvement followed by maintenance of the training benefits.

## Competing interests

The authors declare that they have no competing interests.

## Authors' contributions

LO designed initially the land based perturbation training program. All authors contributed to design of the study and the development of the water based balance training program that include perturbation exercises. IM and LO and OE wrote the paper, OE will administer the training program. IT collects the data and subject recruitment and IM process the data and perform the statistical analyses. All authors contributed significantly to the preparation of the paper, and read and approved the final version.

## Pre-publication history

The pre-publication history for this paper can be accessed here:


